# From repair to reconstruction: a holistic perspective in abdominal wall hernia surgery

**DOI:** 10.3389/fsurg.2026.1782612

**Published:** 2026-04-15

**Authors:** Xin-liang Hou, Ting Zeng, Xu Wang, Li-ye Tan

**Affiliations:** 1 Department of General Surgery, The Second Affiliated Hospital of Mudanjiang Medical University, Mudanjiang, China; 2 Department of Anesthesiology, Hongqi Hospital Affiliated to Mudanjiang Medical University, Mudanjiang, China

**Keywords:** functional reconstruction, hernia, multidisciplinary collaboration, patient-reported outcome, perioperative management, surgery, surgical outcome

## Abstract

The field of abdominal wall hernia surgery is transitioning from a traditional focus on anatomical repair to a more comprehensive model centered on functional reconstruction. This paradigm shift expands the primary goal from mere defect closure to the restoration of abdominal wall integrity, dynamic stability, and physiological function. This perspective article examines this progression and highlights the critical role of integrating functional reconstruction with structured perioperative management to enhance long-term surgical outcomes and patient quality of life. We explore the clinical impact of technical innovations—including minimally invasive component separation, advanced prosthetic materials, and robotic-assisted techniques—alongside the implementation of individualized perioperative care pathways. Multidisciplinary collaboration is emphasized as a foundational framework for delivering personalized treatment. Several challenges remain, including optimal material selection, comparative evaluation of surgical approaches, and health economic assessments. Addressing these issues requires robust prospective studies to strengthen the evidence base. Future directions should prioritize the development of standardized functional assessment tools, the integration of artificial intelligence in surgical planning, and the incorporation of function-oriented principles into surgical education and practice. Through these advancements, abdominal wall hernia surgery can fully evolve into a patient-centered specialty focused on achieving sustainable, long-term benefits.

## 1 Introduction

The surgical management of abdominal wall hernias continues to present significant clinical challenges. Persistently high recurrence rates remain a primary concern despite advancements in operative techniques and prosthetic materials (1, 2). Furthermore, postoperative complications, including chronic pain, intra-abdominal hypertension, and surgical site events, adversely affect both immediate recovery and long-term patient well-being (3, 4). The frequency of these issues highlights the constraints of conventional surgical approaches in securing durable and optimal outcomes (5).

Historically, abdominal wall hernia repair has emphasized anatomical restoration. The traditional paradigm primarily sought the mechanical closure of the fascial defect through sutures or mesh augmentation. While this focus on structural repair is essential, it offers an incomplete perspective (6). It often overlooks the abdominal wall's functional role as a dynamic structure involved in respiration, core stability, and pressure regulation (7). Additionally, this model frequently concentrates on the operative procedure itself, with insufficient integration of the patient's overall health status—such as nutrition, comorbidities like obesity and diabetes, and tissue characteristics—into the treatment plan (8). This narrow, technique-driven focus may contribute to suboptimal long-term results.

A shift in perspective is therefore required to advance the field. Modern hernia surgery is evolving toward a more comprehensive strategy that integrates two key principles. The first is functional restoration, which aims to reestablish not only anatomical integrity but also the muscular and fascial coordination necessary for normal abdominal wall mechanics (9, 10). The second is structured perioperative care, which involves coordinated management across all phases of treatment (11, 12). This includes preoperative optimization of modifiable risk factors, intraoperative decision-making tailored to specific hernia characteristics and patient physiology, and postoperative protocols designed to facilitate recovery and monitor long-term outcomes.

**Figure 1:**
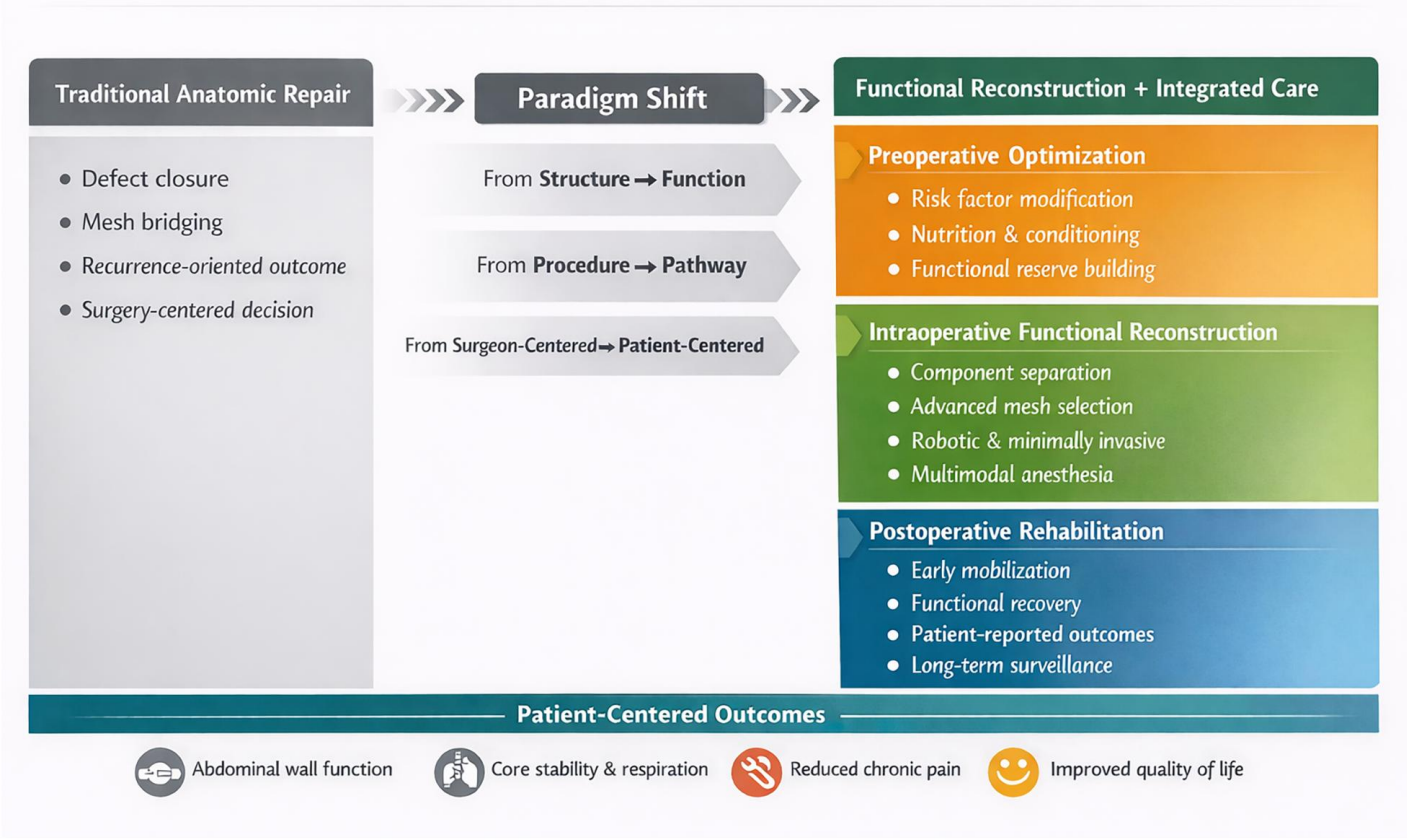
Paradigm shift from anatomic repair to functional reconstruction with integrated perioperative management in abdominal wall hernia surgery.

In summary, abdominal wall hernia surgery is transitioning toward a more holistic, patient-centered model (Figure 1). By combining the goals of functional anatomical reconstruction with rigorous perioperative management, this integrated approach addresses the limitations of earlier methods. It aims to improve durability, minimize complications, and ultimately enhance the patient's functional capacity and quality of life, representing the contemporary direction of the specialty.

## 2 Evolving from anatomic repair to functional reconstruction in abdominal wall surgery

### 2.1 Era of anatomic repair

The traditional paradigm in abdominal wall hernia surgery focused on anatomic restoration. The primary goal was the mechanical closure of fascial defects through direct suture or synthetic mesh implantation (13). Techniques such as the Lichtenstein repair or intraperitoneal onlay mesh placement achieved this by bridging or reinforcing the hernia opening (14). While effective in reducing early recurrence, this approach had significant limitations. It largely treated the abdominal wall as a passive barrier, overlooking its dynamic biomechanical role. Consequently, repair often resulted in reduced abdominal compliance, impaired muscle function, and complications such as chronic pain or increased intra-abdominal pressure (12, 15). Moreover, this model prioritized technical success over the patient's functional recovery and long-term quality of life, reflecting a narrow, procedure-oriented perspective (16) (Table 1).

**Table 1:** Comparison between traditional anatomic repair and modern functional reconstruction with integrated perioperative care.

Domain	Traditional anatomic repair	Functional reconstruction & integrated care
Surgical goal	Defect closure	Restoration of abdominal wall function
View of abdominal wall	Static barrier	Dynamic biomechanical unit
Surgical planning	Technique-driven	Patient- and function-oriented
Perioperative care	Fragmented	Structured, continuous pathway
Outcome evaluation	Recurrence rate	Function + PROs + QoL
Team structure	Surgeon-centered	Multidisciplinary team
Long-term focus	Limited	Long-term functional integrity

PROs, patient-reported outcomes; QoL, quality of life.

### 2.2 Emergence of functional reconstruction

To address these shortcomings, the field has shifted toward a comprehensive strategy of functional reconstruction. This approach aims not only to close the anatomic defect but also to restore the abdominal wall's dynamic stability, muscular coordination, and physiological function in respiration and core movement. Its implementation is supported by parallel advances in surgical techniques and technology.

Importantly, the shift toward functional reconstruction has been accompanied by significant progress in minimally invasive surgery. The transition from traditional open repair to laparoscopic techniques—such as total extraperitoneal and extended totally extraperitoneal approaches—has markedly reduced access trauma, enhanced visualization of key anatomical structures, and enabled earlier patient mobilization (17–19). These technical advances continue to play a crucial role in improving perioperative safety and supporting long-term functional recovery.

Advancements in component separation: Techniques have evolved from open anterior approaches to minimally invasive posterior methods, such as endoscopic or robotic-assisted transversus abdominis release (20, 21). These approaches allow for midline reconstruction with less tissue disruption, better preservation of neurovascular supply, and enhanced recovery of muscular function (22, 23).

Innovations in prosthetic materials: Mesh design has progressed toward lightweight, partially absorbable, and biologic materials (24). These advanced meshes provide adequate mechanical support while facilitating tissue integration and remodeling, thereby promoting a more natural and functional repair with reduced long-term complications (25).

The role of robotic surgery: Robotic platforms enhance precision, visualization, and dexterity (26). This facilitates complex dissection, meticulous suture repair, and accurate mesh placement in challenging anatomic settings, contributing to more reliable and reproducible functional outcomes (27).

Comprehensive outcome assessment: Success is no longer measured by recurrence rates alone (28). Modern evaluation incorporates objective functional metrics—such as core muscle strength and respiratory capacity—alongside validated patient-reported outcomes (7, 29). These tools assess pain, physical function, and quality of life, providing a holistic view of surgical success (30).

This transition from a purely anatomic to a functionally oriented paradigm represents a fundamental evolution in abdominal wall surgery. It redefines the discipline from one focused on defect closure to one dedicated to restoring patient-centered physiological and quality-of-life outcomes.

## 3 Integrated perioperative management: optimizing the entire surgical pathway

Successful outcomes in modern abdominal wall reconstruction depend on more than operative technique alone. They require systematic, coordinated management throughout the entire perioperative period. This integrated approach aims to reduce surgical risks, accelerate functional recovery, and improve long-term results by optimizing care across the preoperative, intraoperative, and postoperative phases, reflecting a comprehensive and patient-centered treatment model.

### 3.1 Preoperative optimization (prehabilitation)

The preoperative phase focuses on prehabilitation, which proactively prepares patients both physiologically and psychologically for surgery (31). It begins with a thorough risk assessment, including detailed evaluation of hernia characteristics and a systematic review of modifiable comorbidities such as obesity, diabetes, and smoking status (32). Based on this evaluation, tailored interventions are implemented. Nutritional support is provided when indicated to enhance tissue repair (33). Patients are instructed in breathing exercises and progressive core strengthening to improve functional reserve (33). Concurrent management of medical comorbidities—such as glycemic control, weight reduction, and smoking cessation—is emphasized (34). Together, these measures aim to improve the patient's baseline resilience, optimize conditions for healing, and increase tolerance to surgical stress.

### 3.2 Intraoperative strategy integration

Intraoperative care is critical for precise anatomical and functional restoration while supporting rapid recovery. Surgical approach selection is individualized, balancing the patient's functional objectives, abdominal wall anatomy, and tissue quality to choose among open, laparoscopic, or robotic-assisted techniques (35). Anesthesia and analgesia are similarly optimized, typically using a multimodal strategy centered on regional nerve blocks (36). This approach provides effective pain control while minimizing the need for systemic opioids, thereby reducing side effects and facilitating earlier postoperative mobilization (37).

Furthermore, surgical quality is fundamentally linked to a thorough understanding of abdominal wall neuroanatomy (38). Meticulous intraoperative nerve identification and preservation are essential components of the functional approach (39). This specific focus on neural integrity serves as a primary mechanism for mitigating the risk of chronic postoperative pain, thereby directly enhancing long-term patient comfort and functional outcomes (40, 41).

### 3.3 Postoperative rehabilitation and long-term management

Postoperative management focuses on consolidating surgical results and ensuring sustained recovery. Early, guided mobilization and structured physical therapy are encouraged to prevent complications, restore abdominal wall function, and support a return to normal activity (42). Crucially, care extends beyond discharge through scheduled long-term follow-up (43). Follow-up assessments monitor not only for hernia recurrence but also for the development of chronic pain, changes in patient-reported quality of life, and the maintenance of functional abdominal wall integrity (44, 45).

By integrating care across all phases of treatment, this model transforms hernia surgery from a discrete procedural event into a coordinated continuum of recovery. It represents a fundamental advance in achieving durable outcomes and improving the long-term well-being of patients undergoing abdominal wall reconstruction.

## 4 Multidisciplinary collaboration: A framework for implementing the modern approach

Successfully implementing the principles of functional reconstruction and integrated perioperative care requires an organized and collaborative framework (46). A multidisciplinary team (MDT) model is central to this approach, integrating expertise from different specialties to guide the patient throughout the entire treatment process—from initial evaluation to long-term recovery (11, 46). However, the extent of MDT involvement should be scaled according to case complexity (46, 47). While comprehensive MDT evaluation is highly beneficial for patients with large defects, loss of domain, significant comorbidities, or prior repair failures, many routine hernia repairs can be managed safely using standardized protocols without formal team discussions (47, 48). Therefore, a stratified implementation model offers a pragmatic and resource-efficient approach.

A comprehensive MDT typically includes a hernia and abdominal wall surgeon, an anesthesiologist, a rehabilitation physician, a nutritionist, a physical therapist, and dedicated nursing staff (49). The surgeon leads the overall treatment strategy, while the anesthesiologist manages pain control and enhances recovery protocols (50). The rehabilitation physician and physical therapist develop tailored exercise programs to restore function (51). The nutritionist optimizes dietary status to support healing, and the nursing team provides continuous education, monitoring, and support (49). This team works together through regular meetings and shared clinical pathways, ensuring coordinated care from assessment through postoperative follow-up.

Crucially, the patient is an active participant in this process. Through clear education and shared decision-making, patients understand their treatment goals and engage in their own recovery (52). This collaborative approach improves adherence to treatment, promotes self-management, and leads to better functional outcomes and quality of life. By embedding teamwork and patient partnership into practice, the MDT model turns contemporary surgical principles into sustainable, patient-centered pathways for care (46).

## 5 Clinical evidence and unresolved issues

### 5.1 Evidence supporting functional reconstruction and integrated management

A growing body of clinical research supports the combined approach of functional reconstruction and comprehensive perioperative care. Studies, including prospective cohorts and randomized controlled trials, show that techniques prioritizing functional recovery—such as posterior component separation—significantly lower recurrence rates in complex ventral hernias (53). This strategy also integrates multimodal pain management, early rehabilitation, and tailored physical therapy (7). These elements collectively reduce chronic postoperative pain and improve patient-reported outcomes in physical function, daily activity, and quality of life (44). The evidence confirms that focusing on both anatomical and functional restoration leads to better overall patient recovery (54).

### 5.2 Unresolved questions and ongoing debates

Despite progress, several clinical questions remain unanswered. First, the optimal choice of mesh material—whether permanent synthetic, absorbable, or biologic—requires long-term comparative data, especially in contaminated surgical fields (54). Second, while minimally invasive approaches offer shorter recovery times, their long-term functional outcomes compared to open techniques need further validation (55, 56). Finally, the cost-effectiveness of integrated multidisciplinary care should be evaluated (46). Although initial costs may be higher, potential savings from reduced recurrences and complications should be assessed through formal health economic studies to justify broader adoption.

## 6 Future directions and the evolution of general surgery

### 6.1 Research priorities

Advancing abdominal wall surgery requires focused research in three key areas. First, there is a need for standardized, validated tools to measure abdominal wall function. These should combine objective metrics—such as core strength and respiratory mechanics—with patient-reported outcomes, providing a comprehensive basis for evaluating recovery (57). Second, artificial intelligence applied to preoperative imaging can enhance surgical planning. Artificial intelligence algorithms may help analyze muscle quality, defect size, and anatomical relationships, supporting more individualized operative strategies (58). Third, regenerative medicine offers a promising long-term direction. Research into bioactive materials that promote functional tissue regeneration, rather than passive repair, could eventually shift the paradigm from structural reinforcement toward biological restoration (13).

### 6.2 Transforming surgical training and practice

Integrating this new approach requires changes in both education and clinical culture. Surgical training programs should formally incorporate the principles of functional reconstruction and integrated perioperative care (59). This includes dedicated teaching on abdominal wall biomechanics, hands-on training in advanced repair techniques, and emphasis on patient-centered outcome measures. Beyond technical skill, a broader cultural shift is needed (60). Clinical discussions, quality assessments, and research evaluations should prioritize long-term functional recovery and quality of life, moving beyond the traditional binary focus on hernia recurrence (44). By embedding these principles in education and practice, the function-oriented model can become the standard of care in modern abdominal wall surgery.

## 7 Summary

Abdominal wall hernia surgery is shifting from a traditional focus on anatomical closure toward a holistic model centered on functional reconstruction and integrated perioperative care. This contemporary approach requires surgeons to consider not only the mechanical repair of the defect but also the restoration of abdominal wall physiology, patient-specific functional goals, and long-term quality of life.

In practice, this model emphasizes multidisciplinary collaboration. Surgeons should lead coordinated care teams—including specialists in anesthesia, rehabilitation, nutrition, and nursing—to develop individualized treatment pathways. The aim is to improve both surgical outcomes, such as reducing recurrence, and patient-centered results, including functional recovery and overall well-being.

The field's continued advancement relies on three interconnected efforts: ongoing innovation in surgical techniques and biomaterials, rigorous clinical research to strengthen the evidence base, and the integration of functional principles into surgical education and guidelines. Together, these will help ensure that abdominal wall hernia surgery evolves in a way that consistently prioritizes lasting patient benefit.

## Data Availability

The original contributions presented in the study are included in the article/Supplementary Material, further inquiries can be directed to the corresponding author.
